# Isolation and Characterization of Circulating Tumor Cells in Squamous Cell Carcinoma of the Lung Using a Non-EpCAM-Based Capture Method

**DOI:** 10.1371/journal.pone.0142891

**Published:** 2015-11-16

**Authors:** Cecilia Bozzetti, Federico Quaini, Anna Squadrilli, Marcello Tiseo, Caterina Frati, Costanza Lagrasta, Cinzia Azzoni, Lorena Bottarelli, Maricla Galetti, Angela Alama, Silvana Belletti, Rita Gatti, Antonio Passaro, Angela Gradilone, Andrea Cavazzoni, Roberta Alfieri, Pier Giorgio Petronini, Mara Bonelli, Angela Falco, Cecilia Carubbi, Giuseppe Pedrazzi, Rita Nizzoli, Nadia Naldi, Carmine Pinto, Andrea Ardizzoni

**Affiliations:** 1 Division of Oncology, University Hospital of Parma, Parma, Italy; 2 Department of Clinical and Experimental Medicine, University of Parma, Parma, Italy; 3 Department of Biomedical, Biotechnological and Translational Sciences, University of Parma, Parma, Italy; 4 Centre for Molecular and Translational Oncology (COMT), Department of Biomedical, Biotechnological and Translational Sciences, Unit of Pathological Anatomy, University Hospital of Parma, Parma, Italy; 5 Lung Cancer Unit, IRCCS AOU S. Martino—IST National Institute for Cancer Research, Genoa, Italy; 6 Division of Thoracic Oncology, European Institute of Oncology, Milan, Italy; 7 Department of Molecular Medicine, Sapienza University of Rome, Rome, Italy; 8 Department of Neuroscience, University of Parma, Parma, Italy; 9 Division of Medical Oncology, S. Maria Nuova Hospital/IRCCS, Reggio Emilia, Italy; 10 Division of Oncology, S.Orsola-Malpighi Hospital, Bologna, Italy; The Ohio State University, UNITED STATES

## Abstract

**Introduction:**

The exclusion of circulating tumor cells (CTCs) that have lost epithelial antigens during the epithelial-to-mesenchymal transition (EMT) process by using Epithelial Cell Adhesion Molecule (EpCAM) based capture methods is still a matter of debate. In this study, cells obtained after depletion procedure from blood samples of squamous cell lung cancer (SQCLC) patients were identified based on morphology and characterized with the combination of FISH assessment and immunophenotypic profile.

**Materials and Methods:**

Five mL blood samples, collected from 55 advanced SQCLC patients, were analyzed by a non-EpCAM-based capture method. After depletion of leukocytes and erythroid cells, the negative fraction was characterized by both FISH using a fibroblast growth factor receptor 1 (FGFR1) probe and by immunocytochemistry. Thirty healthy donors were also tested.

**Results:**

Based on morphology (nuclear dimension ≥10 μm, shape and hypercromatic aspect) suspicious circulating cells clearly distinguishable from contaminant leukocytes were observed in 49/55 (89%) SQCLC patients. Thirty-four of the 44 (77%) samples evaluable for FGFR1 FISH showed ≥ 6 FGFR1 gene copy number on average per cell. Vimentin expression involved 43% (18/42) of pooled circulating SQCLC cells, whereas only 29% (14/48) were EpCAM positive. Confocal microscopy confirmed the localization of FGFR1 probe in suspicious circulating cells. Suspicious circulating elements were also observed in healthy donors and did not show any epithelial associated antigens. A significantly lower number of suspicious circulating cells in healthy donors compared to SQCLC patients was found.

**Conclusions:**

Among the heterogeneous cell population isolated by depletion procedure, the coexistence of cells with epithelial and/or mesenchymal phenotype suggests that EMT may participate to transendothelial invasion and migration of tumor cells in advanced SQCLC. The finding of cells with neither EpCAM or EMT phenotype, retrieved after non-EpCAM-based systems, underlines the presence of suspicious elements in the blood of both SQCLC patients and healthy donors. Further phenotyping and molecular analyses are necessary to fully characterize these circulating elements.

## Introduction

Data from the literature reports that circulating tumor cells (CTCs) may provide important prognostic and predictive information for the treatment of non-small cell lung cancer (NSCLC) patients [1–3]. A major challenge is that a number of approaches have been used for the detection of CTCs, based on their physical and biological characteristics [1–9]. In most studies, technologies used to capture CTCs are based on their recognition by immunomagnetic beads coated with antibodies to Epithelial Cell Adhesion Molecule (EpCAM) and report from 6% to 20% of squamous cell lung cancer (SQCLC) patients with detectable CTCs [1,2]. This low percentage of cases with detectable CTCs may be explained by the fact that EpCAM is not expressed by all CTCs. Indeed, during the epithelial-to-mesenchymal transition (EMT) process, which has been suggested as a crucial step in cancer dissemination, invading mesenchymal tumor cells may loose cell-cell adhesion molecules [4,10–17]. The exclusion of circulating cells with reduced or absent EpCAM expression may prevent the recognition of the overall CTC population. To overcome this limitation, new technologies have been developed to improve detection and characterization of CTCs including immunomagnetic bead separation methods and filter-based size exclusion technologies coupled with cytopathological and molecular approaches [18]. However, regardless of the techniques used to isolate CTCs, data is emerging about doubtful elements that do not fulfil all the criteria accepted for CTC validation, such as round nuclei in cells expressing EpCAM and cytokeratin and lacking the leukocyte common antigen CD45 [5,6,19]. These doubtful elements do not show any immunoreactivity for EpCAM and CD45 and display unusually large size comparable to CTCs. The origin of these elements is still unknown and they may represent a cell fraction common to both patients and healthy donors [6]. The primary objective of this study was to evaluate the feasibility of detecting CTCs in blood samples from advanced SQCLC patients by using a non-EpCAM-based capture method. The characteristics of cells obtained by depletion procedure were documented on the basis of morphology and the combination of fibroblast growth factor receptor 1 (FGFR1) gene assessment with immunophenotypic profile.

## Materials and Methods

### Patients

Fifty five patients with advanced SQCLC attending the Oncology Unit of the University Hospital of Parma, Italy, were prospectively enrolled in this study. This study was conducted according to a protocol approved by the institutional review board/independent ethics committee, and informed consent was obtained from all patients for the use of blood samples and clinical information.

Patients with SQCLC (47 males and 8 females, all smokers) had a mean age of 68 years (range 45–86) with disease stage IV in 41 cases (75%) and III in 14 cases (25%). Blood samples for CTC detection were collected in 37 cases before therapy (26 before first-line, 7 before second-line treatment, 4 before radiotherapy), in 12 cases during chemotherapy (8 during first-line and 4 during second-line) and in 6 cases after chemotherapy (3 after chemo-radiotherapy for stage III disease and 3 after two lines of medical therapy).

A control group of 30 healthy donors was also examined.

### Depletion procedure

Five mL peripheral blood samples were processed for each patient immediately or no later than 4 hours after blood withdrawal. After red blood cell lysis (Red blood cell lysis solution, Miltenyi Biotec, Bergisch Gladbach, Germany), the sample was centrifuged (300 xg) for 10 min and the supernatant was discarded. The cell pellet was magnetically labelled with CD45 Microbeads (Miltenyi Biotec) and CD235A (Glycophorin A) Microbeads (Miltenyi Biotec) for 15 min at 4°C to deplete leukocytes and erythroid cells respectively and loaded in a AutoMACS^®^ separator (Miltenyi Biotec). Columns were processed using the manufacturer’s suggested protocol. The negative fraction was centrifuged (800 xg) for 10 min and the pellet sedimented on a cytology slide, air dried and stored at –20°C until the time of fluorescence *in situ* hybridization (FISH) assay. For immunocytochemistry the cell pellet was fixed and cytocentrifuged. The recovery rate of the depletion procedure was previously described [20].

### EpCAM-based system

CTC analysis was performed using the CellSearch^®^ system (Veridex, Raritan, NJ). Blood samples (7.5 mL) were collected in 10 mL CellSave (Veridex) preservative tubes, stored at room temperature and processed within 96 hours from collection, according to the manufacturer’s instructions [19]. CTCs were defined as cells having a 4’,6-diamidino-2-phenilindole (DAPI) stained nucleus and expressing EpCAM and cytokeratins (8, 18, 19) in the absence of CD45 antigen.

### ScreenCell system

Size-selective isolation was performed using the ScreenCell^®^ device (ScreenCell, Paris, France) from 3 mL whole blood filtered through a microporous membrane filter containing 8 μm pores.

The samples were processed within 4 hours after blood collection. The blood was diluted with 4 mL of red blood cell lysis buffer (ScreenCell) and incubated for 10 min at room temperature. The samples were filtered in a few seconds through the porous membrane. After rinsing with sterile phosphate-buffered saline (PBS 1X) solution, filters were counterstained with haematoxylin and eosin, air dried and mounted on a glass slide using Faramount mounting medium (DAKO Glostrup, Denmark), for evaluation by light microscopy. Suspicious circulating cells (epithelial and mesenchymal) were defined according to the following criteria: cell diameter approximately twice that of the filter pore (8μM), high nuclear-cytoplasmic ratio (> 0.5), nuclear pleomorphism, nuclear hyperchromatism and number of nucleoli.

### *FGFR1*- Fluorescence *in situ* hybridization (FISH)

*FGFR1* gene copy number was assessed by FISH using a break-apart *FGFR1*/Chromosome 8 Centromere (CEN8) FISH probe set (Cytocell Aquarius, Cambridge, UK). Unstained cytology slides obtained after the depletion procedure were fixed in absolute methanol and incubated in wash buffer (0.3% NP-40, 2 × saline–sodium citrate buffer, pH 7.0–7.5) at 37°C for 30 min. After alcohol dehydration, slides were hybridized at 67°C for 5 min using a Hybrite denaturation/hybridization system for FISH (Abbott-Vysis, Wiesbaden, Germany) and incubated overnight at 37°C. The following day, slides were incubated in wash buffer at 72°C for 2 min, air dried in the dark and counterstained with DAPI (Abbott-Vysis).

FISH images were processed utilizing a Nikon Ni-U (Nikon Corporation, Japan) fluorescence microscope equipped with a 100-W mercury lamp. Separate narrow band pass filters for the detection of spectrum red, green, aqua and DAPI were used. Each sample was entirely scanned for both the morphologic identification of cells based on nuclear DAPI staining and for *FGFR1* gene copy number assessment. Separate image acquisitions were made on each field for red, green and aqua signals at 11 different levels for each cell. Only distinct fluorescent signals distributed at different levels of the cell were counted. Cases showing two red/green signals relative to *FGFR1* gene and two aqua signals relative to CEN8 per cell were classified as disomic. Cases with a ratio *FGFR1*/CEN8 signals ≥ 2 and cases with ≥ 6 *FGFR1* signals on average per cell were defined as amplified [21].

Cultured H460 and H520 NSCLC cell lines were used as control for *FGFR1* diploid asset and amplification, respectively [22]. Cancer cell lines derived from the American Type Culture Collection (ATCC) and were cultured as recommended.

### Immunocytochemistry

After the depletion procedure, the cytocentrifuged preparations were processed for immunocytochemistry to document the expression of antigens listed in Table 1.

**Table 1:** Antibodies and their dilutions used for immunocytochemistry.

Antigen	Company	Primary Antibody	Clone	Dilution	Secondary Antibody
**EpCAM**	Abcam	Rabbit monoclonal	E144	1:50	Anti Rabbit FITC
**FGFR1**	Cell Signaling Technology	Rabbit monoclonal	D8E4XP	1:100	Anti Rabbit FITC
**Vimentin**	Merck Millipore	Mouse monoclonal	VIM3B4	1:100	Anti Mouse FITC
**CD41**	Abbiotec	Mouse monoclonal	HIP11	1:100	Anti Mouse FITC
**CD45**	DAKO	Mouse monoclonal	2B11+PD7/26	1:100	Anti Mouse FITC
**CD68**	Novus Biological	Mouse monoclonal	KP1	1:20	Anti Mouse FITC
**CD31**	DAKO	Mouse monoclonal	JC70A	Ready-to-use	Anti Mouse FITC
**CD133**	Merck Millipore	Mouse monoclonal	17A6.1	1:20	Anti Mouse FITC
**CD117**	DAKO	Rabbit polyclonal	_	1:20	Anti Rabbit FITC
**Pan Cytokeratin**	DAKO	Mouse monoclonal	AE1/AE3	1:50	Anti Mouse FITC

FITC, Fluorescein isothiocyanate.

To this purpose, the pellet obtained after depletion procedure was immediately fixed in 4% paraformaldehyde for 20 min and cytocentrifuged. Cytospin preparations were incubated with the primary antibody and then with specific fluorochrome-conjugated secondary antibody (Sigma Aldrich, St. Louis, MO) for the evaluation of immunophenotypes by fluorescence microscopy (Leica DMI6000B Microsystems, Wetzlar, Germany). Nuclei were counterstained with DAPI (Sigma) and coverslips mounted with Vectashield^®^ mounting medium (Vector Laboratories, Burlingame, CA).

H1703 and human bone marrow stromal adherent (hBM-StCs) cell lines were used as positive control for FGFR1 and vimentin expression, respectively. H1703 cell line was used as positive control for EpCAM expression and a human erythroleukemia cell line (HEL) with megakaryocyte properties for CD41 expression. Negative controls were represented by omitting the primary antibody from the immunereaction of each tested antigen. All microscopic images were acquired using the same setting in term of time and gain of exposure.

### Confocal microscopy

After removal of the coverslip, slides previously tested by FISH and immunocytochemistry were washed in PBS and incubated with propidium iodide (PI) for 5 min. After washing with PBS, slides were mounted with Vectashield^®^ (Vector Laboratories) mounting medium and stored at 4° C until confocal analysis. The confocal system employed in this study was the LSM 510 Meta scan head integrated with the Axiovert 200 M inverted microscope (Carl Zeiss, Jena, Germany). Specimens were observed through a 63x, 1.4 NA oil immersion objective. FITC and PI were excited with 488 nm argon and 543 nm He-Ne laser lines, respectively. Image acquisition was carried out in a multitrack mode, with the relevant beamsplitters; barrier filters were 505–530 band pass and 560 long pass for the above signals, respectively. A series of x-y sections was acquired with a z-step of 0.5 μm, to cover the whole height of the samples.

### Statistics

Statistical calculations were performed by IBM-SPSS v 20. The two groups, patients and healthy donors, were compared for central tendency by Student’s t-test and the non parametric Mann-Whitney test. Sensitivity and specificity were investigated by ROC analysis.

## Results

### Cells isolated by depletion procedure from SQCLC patients

#### Morphology

**Fig 1:**
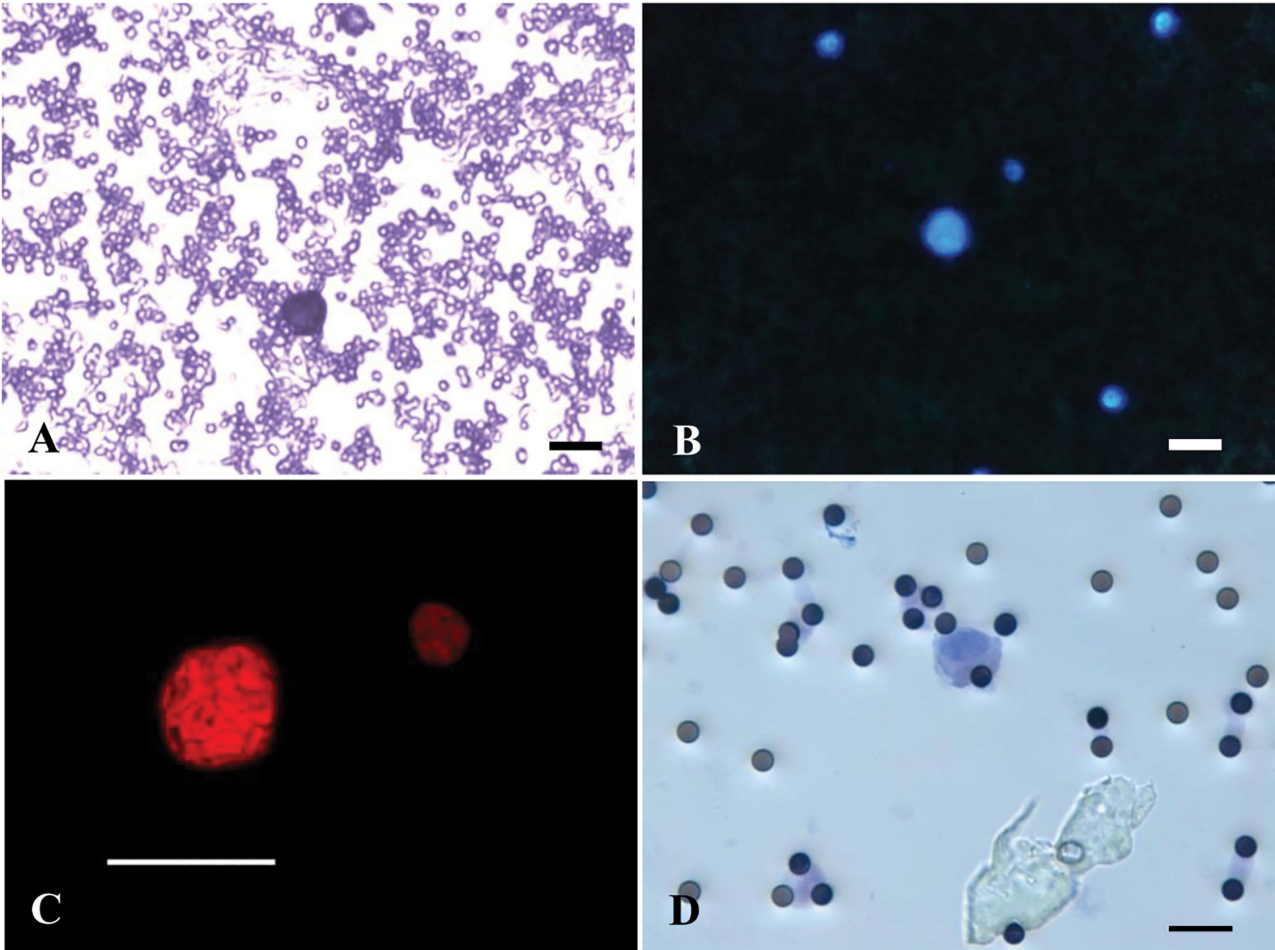
Suspicious circulating elements in SQCLC patients. Representative image of a suspicious circulating element (SCE) isolated by depletion procedure and stained by hematoxylin; contaminant platelets and red blood cell ghosts are present in the background (A). DAPI stained SCE nucleus and small contaminant white blood cells (B). Confocal image of a large hyperchromatic SCE nucleus labelled by the red fluorescence of propidium iodide (PI); a small PI stained nucleus of a white blood cell is also present (C). Weak hematoxylin and eosin stained nucleus of a SCE isolated by the ScreenCell device. Black round dots correspond to 8 μm pores of the filter (D). Scale bars A, B, C, D = 20 μm.

Cells clearly distinguishable from contaminant leukocytes were observed in cytological preparations obtained after depletion procedure in 49 of the 55 (89%) SQCLC patients. These cells, that we defined as suspicious circulating elements (SCEs), were recognizable based on distinctive morphologic features *i*.*e*. unusually large size and hyperchromatic aspect of the nucleus as documented in Fig 1.

Nuclear diameter measured on 50 randomly selected DAPI stained nuclei obtained from 5 SQCLC patients ranged from 10.40 to 22.10 μm (mean value ± standard deviation (SD): 14.61 ± 2.30). In each sample, the presence of contaminating leukocytes was used as negative control by virtue of their small size and distinct nuclear morphology. Nuclear diameter measured on 50 randomly selected DAPI stained leukocytes ranged from 4.63 to 7.66 μm (mean ± SD: 6.38 ± 0.79). Based on nuclear dimension (≥10 μm), shape and hyperchromatic aspect, we found that SCEs were present in 49 of the 55 (89%) SQCLC samples subjected to the depletion procedure. Forty percent (22/55) of patients showed more than 10 SCEs/5 mL blood and 7% (4/55) more than 30. The number of SCEs recovered in 55 SQCLC patients ranged between 1 SCE/5 mL blood and 54 SCEs/5 mL blood.

Twenty five of the 55 SQCLC samples assayed by depletion were also tested by the CellSearch system and only two (8%) were found CTC positive fulfilling all Veridex criteria (Table 2). Of the two CTC positive cases detected by the EpCAM-based system, one had 11 CTCs/7.5 mL and the other four CTCs/7.5 mL peripheral blood and both cases resulted positive by depletion procedure having 13 and 14 SCEs/5 mL blood, respectively.

**Table 2:** Comparison between depletion procedure and EpCAM-based enrichment system in 25 advanced SQCLC patients.

	Cell count					
	0	≥1	≥2	≥10	≥20	≥30
	n(%)	n(%)	n(%)	n(%)	n(%)	n(%)
**Depletion procedure (n = 25)**	3 (12)	22 (88)	21 (84)	11 (44)	4 (16)	3 (12)
**EpCAM-based system(n = 25)**	22 (88)	3 (12)	2 (8)	1 (4)	0	0

EpCAM, epithelial cell adhesion molecule.

One blood sample, considered positive by depletion procedure, was tested in parallel by the ScreenCell^®^ device and was judged positive showing elements with a morphology compatible with CTC criteria described in Materials and Methods.

#### *FGFR1* FISH

**Fig 2:**
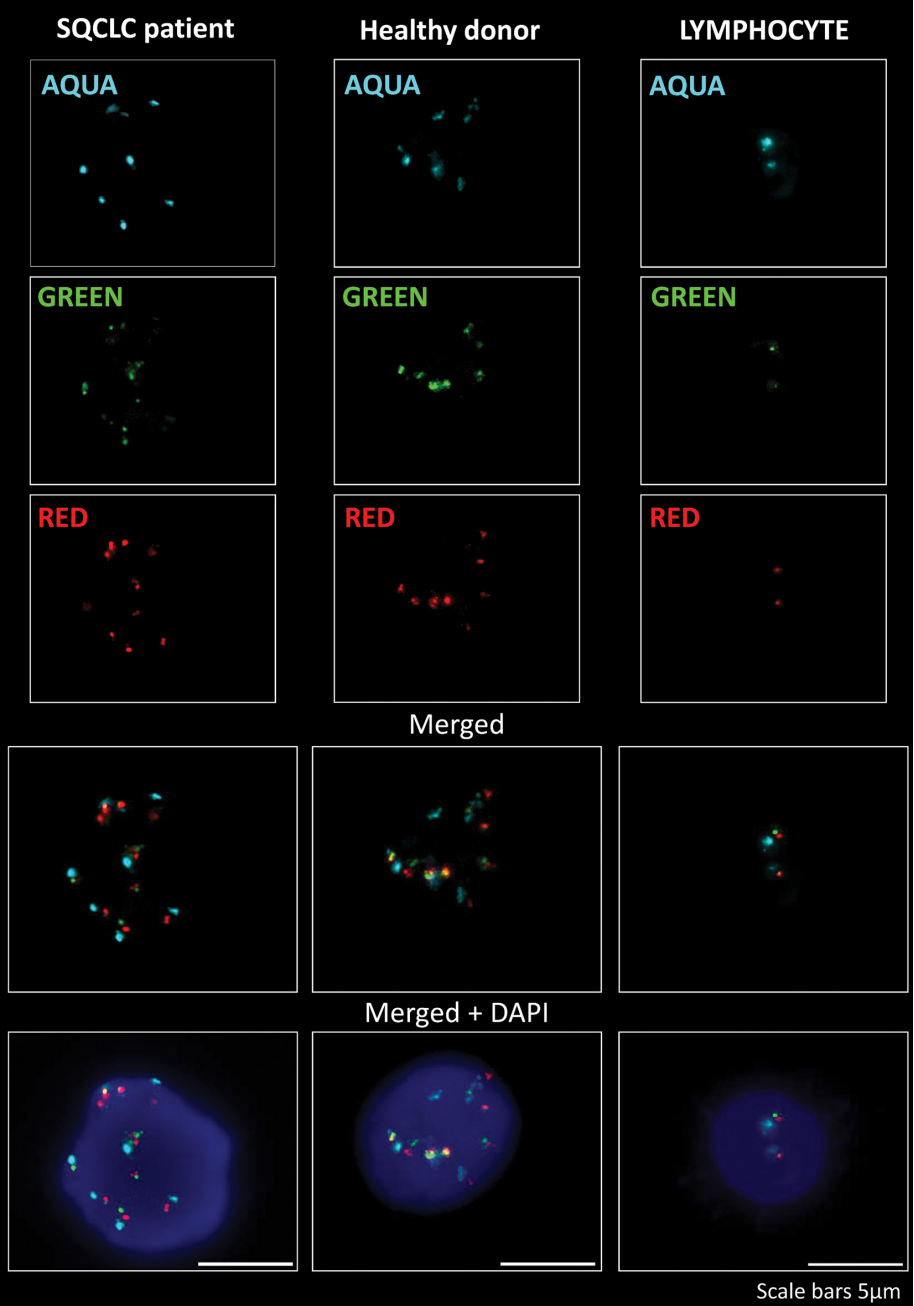
FISH analysis of *FGFR1* gene copy number in suspicious circulating elements. Separate acquisitions of aqua signals corresponding to chromosome 8 centromere and green and red signals corresponding to *FGFR1* gene copy number in a SQCLC patient and a healthy donor are shown. Aqua, green and red signals are merged alone or in combination with DAPI staining of nuclei. A lymphocyte with normal diploid *FGFR1* asset is also shown. Blue fluorescence corresponds to DAPI staining of nuclei. Scale bars = 5 μm.

Of the 49 SCE positive samples tested by depletion procedure, 44 were evaluable for *FGFR1* gene copy number assessment by FISH, while five were not included due to lack of hybridization. The average *FGFR1* gene copy number per nucleus ranged from 2 to 17 (mean ± SD: 6.8 ± 2.8). Thirty four of the 44 (77%) samples evaluable for *FGFR1* FISH had ≥ 6 *FGFR1* signals on average per nucleus. Signals relative to *FGFR1* gene copy number per nucleus were superimposable to the number of CEN8 signals in all samples. An example of a nucleus from a SQCLC patient showing increased *FGFR1* gene copy number is shown in Fig 2.

**Fig 3:**
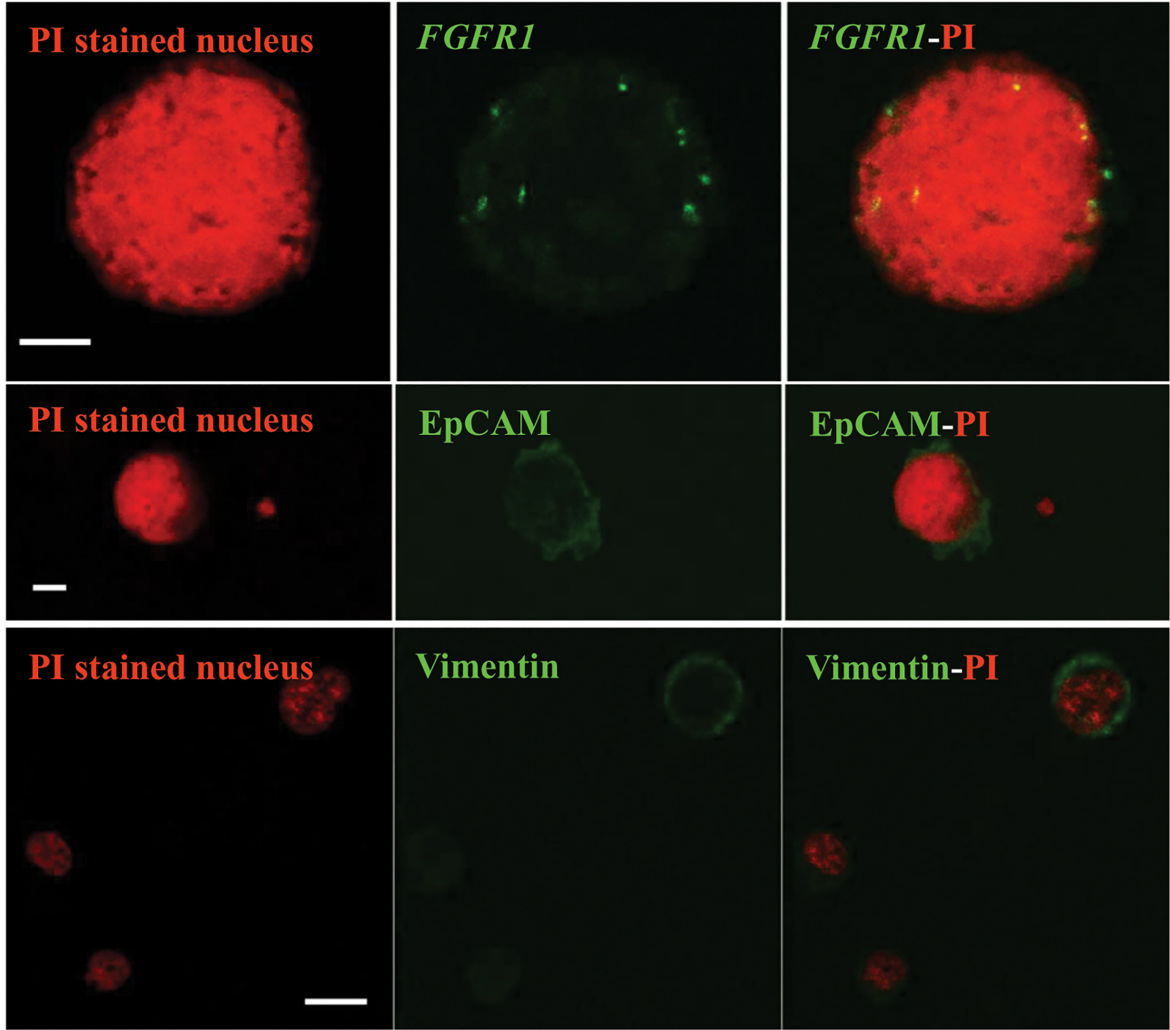
Confocal images of propidium iodide (PI) stained suspicious circulating elements obtained from SQCLC patients. The upper part of the figure shows a PI stained SCE with multiple green signals referring to multiple copies of *FGFR1* gene (scale bar = 5 μm); the middle part shows a PI stained nucleus with EpCAM expression (scale bar = 5 μm) and the bottom part a PI stained nucleus with vimentin expression (scale bar = 10 μm) associated with two small leukocytes showing a faint cytoplasmic fluorescence.

In order to demonstrate that multiple copies of *FGFR1* gene observed in SCEs could not be ascribed to a cellular syncytium, confocal images were obtained and confirmed that acquired images referred to a single cell. A PI stained nucleus with multiple copies of *FGFR1* gene of a SQCLC case is shown in the upper part of Fig 3.

#### Immunophenotype

**Fig 4:**
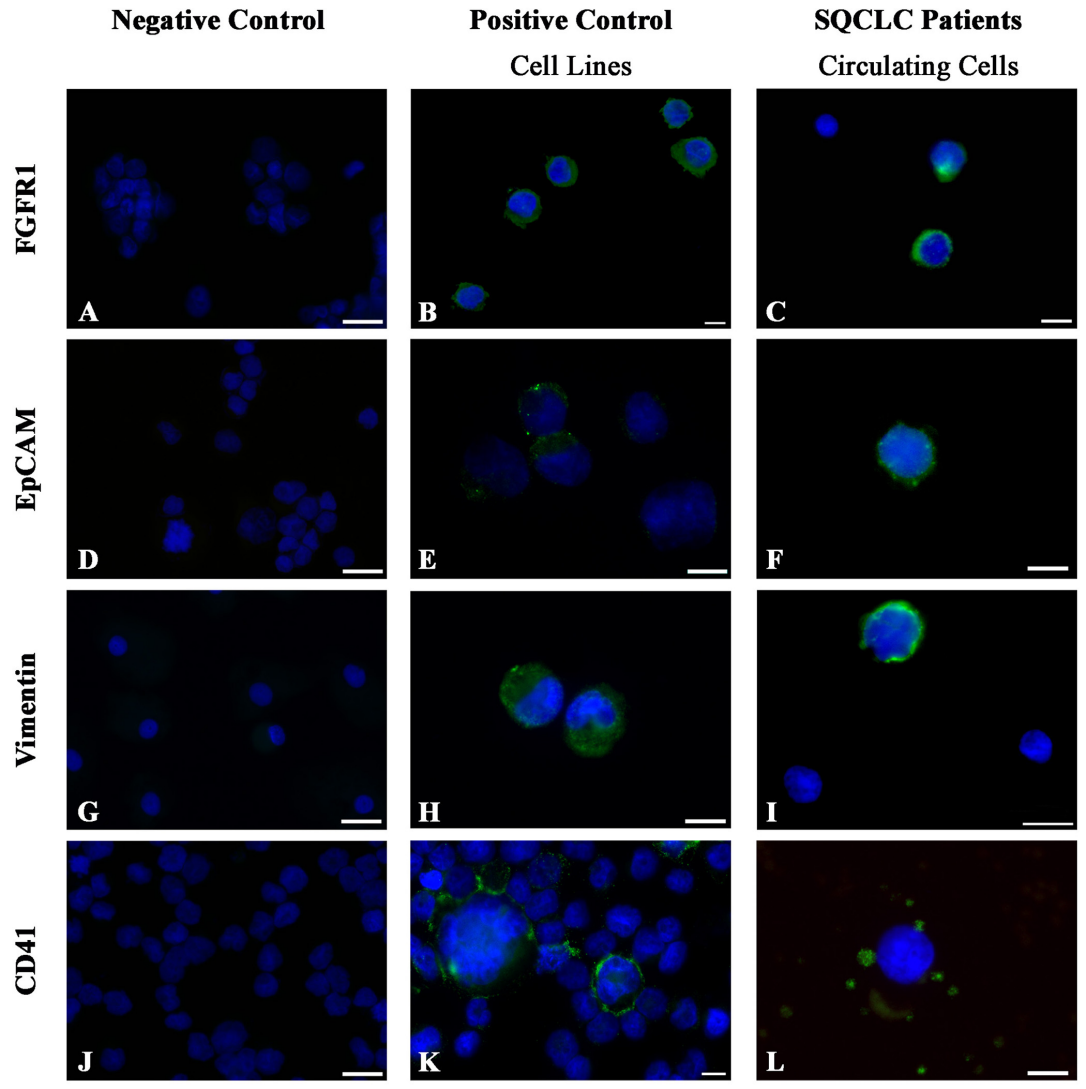
Immunocytochemical characterization of suspicious circulating elements obtained after depletion procedure from SQCLC patients. Images of cytospin preparations for each tested antigen are shown. All antigens were detected by immunofluorescence (green, FITC) and tested in control cell lines in the absence (negative control) or presence of the primary antibody (positive control) and in suspicious circulating elements (SCEs). FGFR1 negative control (A), FGFR1 positive H1703 cell line (B) and FGFR1 positive SCEs (C). EpCAM negative control (D), EpCAM positive H1703 cell line (E) and an EpCAM positive SCE (F). Vimentin negative control (G), vimentin positive hBM-MSC cell line (H) and a vimentin positive SCE (I). CD41 negative control (J), CD41 positive HEL cell line (K) and a CD41 negative SCE (L). Small green fluorescent dots in figure L correspond to platelets. In figures C and I, DAPI stained nuclei of cells showing no immunoreactivity were considered as leukocytes in virtue of their small size. Scale bars: A, D, G and J = 20 μm; B, C, E, F, H, I, K and L = 10 μm.

To determine whether multiple copies of *FGFR1* gene were coupled with the presence of the corresponding protein at cellular level, FGFR1 expression on SCEs was evaluated by immunocytochemistry. The squamous carcinoma cell line H1703 was used as positive control (Fig 4B).

The surface expression of FGFR1 was documented on cytospin preparations of SCEs obtained from 7 randomly selected SQCLC patients. Although it cannot be excluded that this receptor may be expressed on normal circulating cells, 95% (80/84) of pooled SCEs showed prominent immunofluorescent signals (Fig 4C).

To further document the immunophenotype of SCEs and their potential involvement or dependence on EMT process, the presence of EpCAM and vimentin was determined in four randomly selected SQCLC patients with > 10 SCEs/5 mL blood. Three of the four cases tested for EpCAM showed a surface expression by immunofluorescence, indicating the epithelial origin of cells. Positive immunoreaction of EpCAM positive H1703 cell line and of a SCE is shown in Fig 4E and 4F, respectively. The immunocytochemical analysis of vimentin on the same cell preparations documented the cytoplasmic expression of the intermediate filament in all four examined cases. Positive vimentin staining of hBM-MSCs cell line and of SCEs from a SQCLC patient is shown in Fig 4H and 4I, respectively. Interestingly, vimentin expression involved 43% (18/42) of pooled circulating SQCLC cells, whereas only 29% (14/48) were EpCAM positive. The middle part and the bottom part of Fig 3 document confocal images of an EpCAM positive and a vimentin positive SCE, respectively.

To further document that epitopes involved in EMT were expressed in circulating cells expressing multiple *FGFR1*/CEN8 signals, vimentin was tested by immunocytochemistry on the same slides tested for *FGFR1* FISH. The cytoplasmic staining of vimentin was associated with multiple CEN8 signals (S1 Fig).

CD41 antibody was tested on SCEs isolated by the depletion procedure in order to exclude their megakaryocytic phenotype. As depicted in Fig 4K, the CD41-positive HEL cell line showed typical membrane immunoreactivity. SCE from a SQCLC patient did not display any surface expression of CD41 that conversely labelled few platelets (Fig 4L). In addition, SCEs did not show CD45 labeling whereas CD31 and CD68 positivity was occasionally detected (data not shown).

The average cell diameter ± SD of pooled SCEs tested by immunocytochemistry was 25.41 ± 1.37 μm.

### Cells isolated by depletion procedure from healthy donors

**Fig 5:**
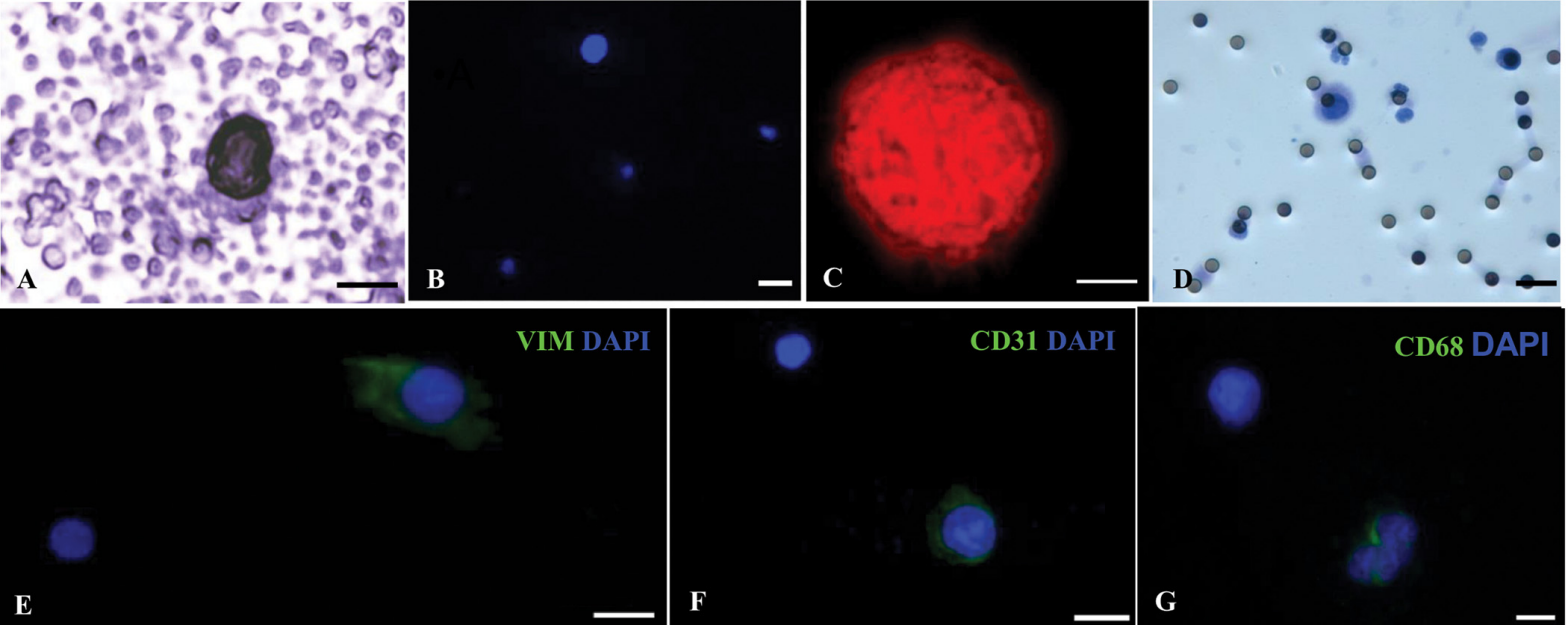
Morphologic and immunophenotypic characteristics of suspicious circulating elements in healthy donors. Representative image of a hematoxylin stained nucleus isolated by depletion procedure (A). A DAPI stained nucleus isolated by depletion procedure and small contaminant white blood cells (B). Confocal image of a propidium iodide (PI) stained nucleus obtained by depletion procedure (C) and a hematoxylin and eosin stained nucleus isolated by the ScreenCell device (D). E—G: cytospin preparations obtained after depletion procedure and tested by immunocytochemistry showing a vimentin positive cell associated with a negative leukocyte (E), a CD31 immunostained cell with a negative leukocyte (F) and two CD68 positive and one CD68 negative cells (G). Scale bars: A, E, F, G = 10 μm; B, D = 20 μm; C = 5 μm.

Twenty eight of the 30 (93%) healthy donors tested by depletion procedure showed more than one suspicious cell/5 mL blood having morphologic characteristics similar to patient SCEs (Fig 5A–5D).

Seventeen percent (5/30) of healthy donors showed more than 10 SCEs/5 mL blood and none more than 22. The mean diameter obtained from 50 pooled nuclei from three healthy donors was 15.17 ± 2.72 μm (range: 10.2–21.5). In order to compare the results obtained by the depletion procedure with those obtained by filtration, five samples tested by depletion were also tested by the ScreenCell device. Four of the five samples were judged positive for the presence of suspicious elements by both methods and one sample was judged negative by both methods by two observers in blind. Suspicious elements isolated by both depletion and filtration showed similar morphologic characteristics with respect to nuclear dimension, pleomorphism and hyperchromatism. Mean diameter obtained from 10 pooled nuclei isolated by the ScreenCell device from three healthy donors was 19.77 ± 3.0 μm (range: 14.5–24.5).

*FGFR1* FISH was evaluable in 16/28 healthy donors and 13/16 had ≥ 6 *FGFR1* signals on average per cell. An example of a nucleus displaying increased *FGFR1* copy number is shown in Fig 2.

Cytospin preparations were obtained from four healthy donors and processed for immunocytochemistry. Among pooled cells with a nuclear diameter ≥ 10 μm none showed EpCAM immunoreaction while vimentin was expressed in 6% of cells (Fig 5E). CD31 and CD68 expressions were found in 16.6% and 17.5% of pooled cells respectively (Fig 5F and 5G) pointing out the presence of a heterogeneous population with endothelial and macrophagic features. No immunoreaction was found for PanCK, CD41 and for the stem cell markers CD133 and CD117.

### Comparison between the number of SCEs detected in patients and healthy donors

**Fig 6:**
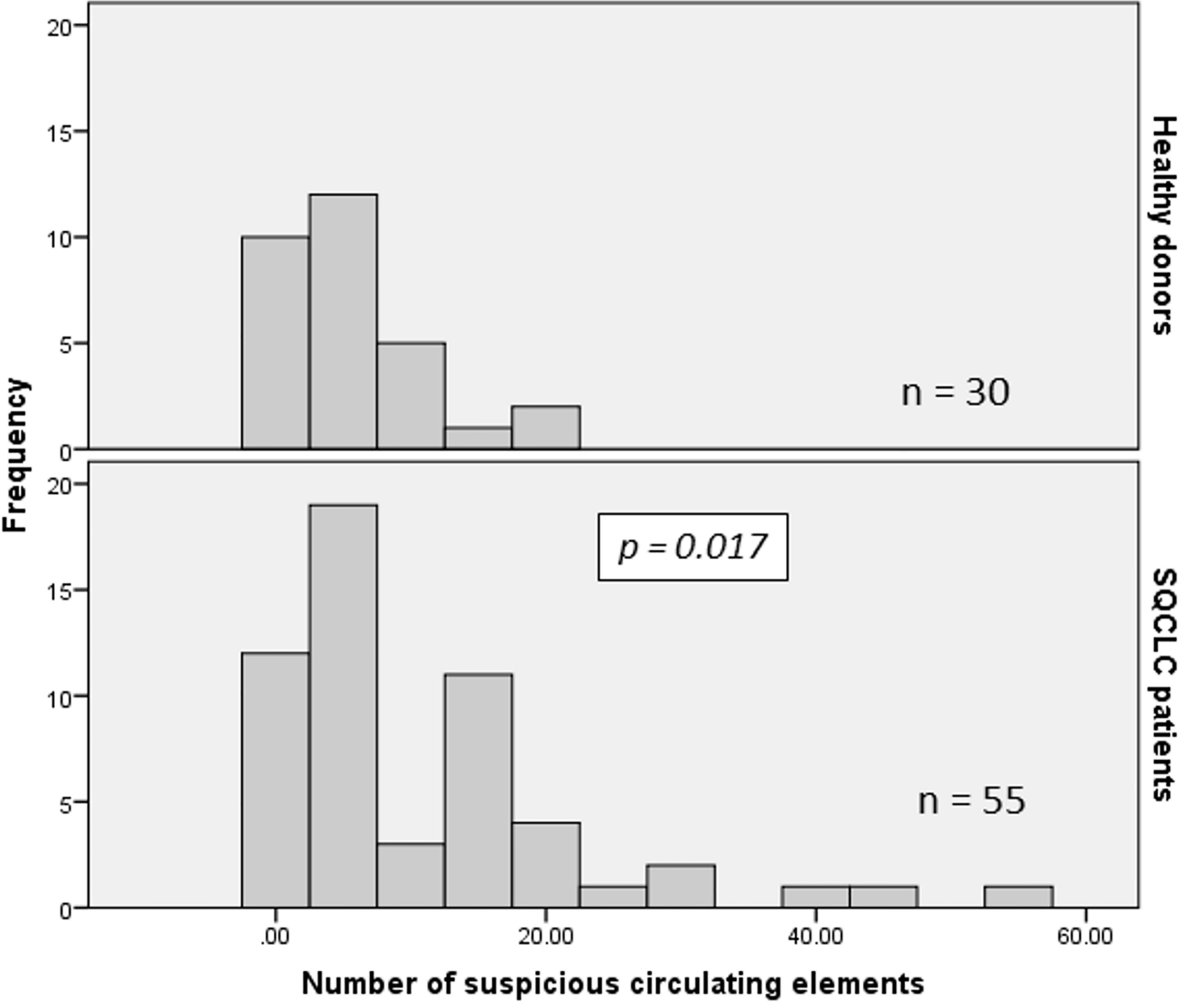
Distribution of suspicious circulating elements isolated by depletion procedure from SQCLC patients and healthy donors.

A statistically significant difference (p = 0.017) was found between the number of SCEs detected in the 55 SQCLC patients (mean ± SD: 10.76 ± 11.40, range 0–54) and in the 30 healthy donors (mean ± SD: 5.37 ± 5.54, range 0–22) (Fig 6; S2 Fig).

By using ROC analysis, we decided to choose 100% specificity that corresponded to 22 SCEs/5 mL blood. Sensitivity at this cut-off value was 11%.

## Discussion

Data from literature reports that during the EMT process cells may loose adhesion molecules and acquire a mesenchymal phenotype [4,10–17], leading to an absent or insufficient EpCAM expression for their detection through EpCAM-based technologies. In our study, this phenomen may justify the prevalence of CTC negative cases tested by the Veridex system in comparison with the depletion procedure. Moreover, our finding of circulating cells with positive vimentin expression supports the contention that EpCAM-based CTC isolation may underestimate the number of neoplastic elements likely delivered from primitive tumor to the blood stream. The expression of mesenchymal associated antigens on SCEs, at least as detected by immunocytochemistry, advances the hypothesis that EMT may participate to transendothelial invasion and migration of tumor cells in advanced SQCLC patients. The identification of SCEs by depletion was documented by their nuclear morphology together with the combination of FISH assessment of *FGFR1* and immunophenotypic characteristics. Confocal images confirmed that: 1) PI-stained nuclei of SCEs were larger than normal blood cells; 2) multiple FISH signals referred to a single nuclear structure; 3) cell membrane or cytoplasmic structures were targeted by EpCAM and vimentin antibodies, respectively. The coexistence of elements with epithelial and/or mesenchymal phenotype, underlines a high heterogeneous pattern within the same patient that could not be identified by EpCAM-based systems. Moreover, EpCAM negative and CD45 negative suspicious elements, with neither endothelial nor macrophagic phenotypes, and morphologically compatible with CTC characteristics were found in both cancer patients and healthy donors.

Previous enumeration of CTCs from NSCLC patients, using EpCAM-based immunomagnetic techniques, reported percentages of CTCs ranging between 6% and 20% using a cut-off **≥** 2 CTCs in 7.5 mL of blood. Data by Allard et al. [19] showed that 20% of patients with metastatic lung cancer had **≥** 2 CTCs in 7.5 mL of blood although the histological subtype was not specified. In a series of 22 primary SQCLC, Tanaka et al. [2] found 18% of the cases with **≥** 2 CTCs, however only 4.5% had **≥** 5 CTCs/7.5 mL of blood. In a more recent work, Krebs et al. [1] found that in a subgroup of 32 SQCLC patients, 6% of the cases had **≥** 2 CTCs/7.5 mL of blood and that the squamous phenotype was associated with fewer CTCs, although they ascribed this finding to the prevalence of patients with stage III disease.

Independently from the methodology used to isolate CTCs, data is emerging about the presence of doubtful elements among the population of hypothetical CTCs. In a study conducted on few NSCLC cases [23], circulating tumor microemboli, analysed by a filter-based size exclusion approach (ISET) and by the CellSearch system (Veridex), were exclusively detected by ISET. These contrasting results have been attributed to a defective expression of EpCAM on tumor cells leading to their escape from immunomagnetic trapping. These authors suggested a potential underestimation of tumor cell burden in the circulation of lung cancer patients by approaches using positive selection for epithelial markers, and proposed to combine marker dependent and independent methods. An additional work [5], conducted on 37 patients with lung adenocarcinoma, potential neoplastic elements not fulfilling all Veridex criteria were recovered in 33 of the 37 cases from the Veridex cartridge. These elements included “suspicious objects” and large naked nuclei.

In our experience, suspicious elements with a nuclear diameter >10 μm and nuclear shape comparable with those of CTCs have been also found in samples from healthy subjects. These cells did not show any immunoreaction to epithelial and stem cell antigens and coexisted with cells positive for endothelial or macrophagic markers. These findings suggest the contamination, among the recognized CTC population, of elements of uncertain origin in both cancer patients and healthy donors. In samples obtained by the ISET system, these doubtful elements could be identified as authentic CTCs, based on morphology only. In addition, distinguishing real CTCs from cells greater than 8 μm, like macrophages or endothelial cells only by morphology may be questionable and need to be further investigated. This hypothesis is supported by our finding of SCEs in blood samples concomitantly tested by depletion and filtration. Our observation sustains the notion that a fraction of cells common to both cancer patients and healthy donors may actually exist, and that the definition of genuine CTCs cannot disregard immunophenotypic characterization. In a recent paper by Lin et al [6] both EpCAM+/CD45- and EpCAM-/CD45- elements were isolated in both cancer patients and healthy controls with a high prevalence in patients. However, no further characterization was reported.

Recent studies have identified FGFR1 as a potential therapeutic target in SQCLC and have also demonstrated that amplification rather than mutation, represents the preferred mechanism of FGFR1 activation in this type of tumor [22,24,25]. FGF-FGFR autocrine pathway has been described to dominate in NSCLC cell lines of squamous and large cell carcinoma histologies that more frequently exhibit mesenchymal transition [25,26]. The evidence that *FGFR1* gene copy number is increased in our series of NSCLC patients points out the contention that *FGFR1* asset may be related to EMT phenotype supporting the results recently reported for bladder cancer where activation of FGFR1 was demonstrated to induce EMT in urothelial carcinoma cell lines [27].

Recent data by Pailler et al. [7] reported an elevated numerical chromosomal instability (CIN) of CTCs isolated by ISET in four cases of *ROS1*-rearranged NSCLC patients. These CTCs, further characterized by using a multi-FISH assay to enumerate chromosomes 13, 18, 21, X and Y, showed a high level of aneuploidy. In our opinion and based on our results, this observation underlines the need of further studies to investigate the genomic asset of CTCs isolated by ISET procedures or other non-EpCAM-based systems.

To our knowledge only few studies [4–9] have been published on the biological characterization of CTCs from NSCLC and none on SQCLC patients. The unselected SQCLC patient population together with the low number of healthy donors and the descriptive nature of the study represent the major drawbacks of our investigation. The identification and characterization of a heterogeneous cell population, isolated by depletion procedure, with epithelial or mesenchymal phenotype, could be regarded as particularly favourable for a better comprehension of the dissemination process and mechanisms underlying drug resistance. The finding of ambigous elements underlines that cells with neither EpCAM nor EMT phenotype may be present in both patients and healthy donors and could be isolated by non-EpCAM-based systems. Improvement in capture technologies are required for further phenotyping and molecular analyses aimed to elucidate the origin of these suspicious elements.

## Supporting Information

S1 Fig Cytoplasmic vimentin immunostaining (green fluorescence) and multiple copies of chromosome 8 (aqua signals) in a DAPI stained nucleus of a suspicious circulating element from a squamous cell lung cancer patient (Scale bar = 10 μm). (TIF)

S2 Fig Distribution of suspicious circulating elements isolated by depletion procedure from squamous cell lung cancer patients and healthy donors. (TIF)
