# Supplementary figures and images for: Isolation and Characterization of Circulating Tumor Cells in Squamous Cell Carcinoma of the Lung Using a Non-EpCAM-Based Capture Method
Source: PLoS One. 2015 Nov 16;10(11):e0142891. doi: 10.1371/journal.pone.0142891 (PMC4646671; doi:10.1371/journal.pone.0142891)

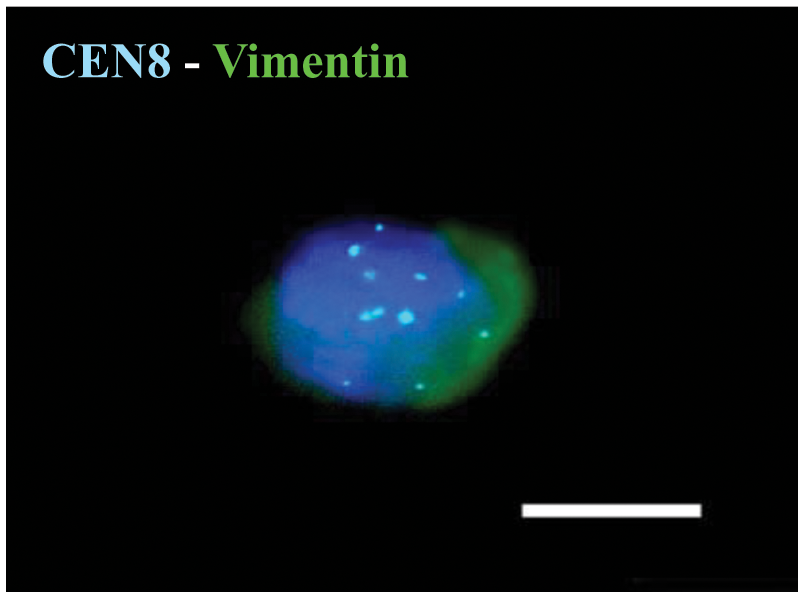

Supplement: S1 Fig — (TIF) [file pone.0142891.s001.tif]

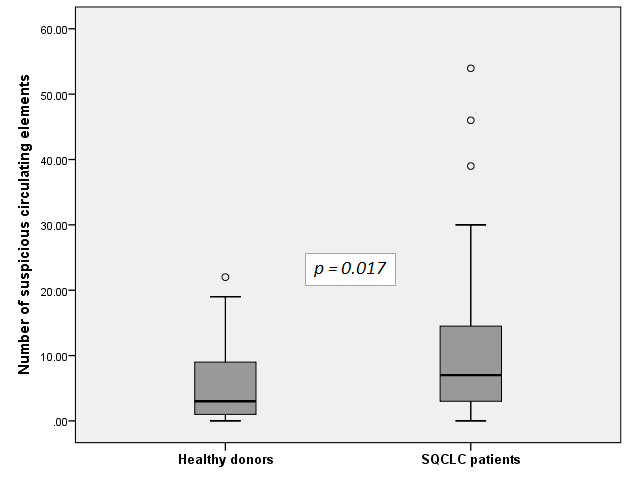

Supplement: S2 Fig — (TIF) [file pone.0142891.s002.tif]
